# Double Lateral Flow Test System for Simultaneous Immunodetection of Enantiomeric Forms of Antibiotics: An Ofloxacin Case Study

**DOI:** 10.3390/bios15120765

**Published:** 2025-11-21

**Authors:** Olga D. Hendrickson, Nadezhda A. Byzova, Anatoly V. Zherdev, Boris B. Dzantiev

**Affiliations:** A.N. Bach Institute of Biochemistry, Research Center of Biotechnology of the Russian Academy of Sciences, Leninsky Prospect 33, 119071 Moscow, Russia; odhendrick@gmail.com (O.D.H.); nbyzova@inbi.ras.ru (N.A.B.); zherdev@inbi.ras.ru (A.V.Z.)

**Keywords:** ofloxacin, S-enantiomer, R-enantiomer, fluoroquinolone, antibiotic, double lateral flow immunoassay, enantiospecific detection, food safety

## Abstract

Antibiotic stereoisomers as components of medicines are typically characterized by different biological activities. Because pharmaceuticals can include a racemic mixture of stereoisomers, monitoring of all forms is required. One contaminant of food products, antibiotic ofloxacin (OFL), as a chiral compound, has two enantiomers—the biologically active S-isomer and less active R-isomer. In this study, a sensitive immunochromatographic test system for simultaneous enantiospeсific detection of the two OFL isomers was developed for the first time. For this, polyclonal antibodies were produced, and conditions for a double lateral flow immunoassay (LFIA) were selected and optimized so that the cross-reactivity with another enantiomer was negligible. The LFIA was performed in a competitive format with gold nanoparticles as a label for secondary antibodies. The achieved LODs/cutoffs were 0.001/10 and 0.007/30 ng/mL for S-OFL and R-OFL detection, respectively; the assay procedure took only 15 min. A double LFIA was performed to detect S-OFL and R-OFL in milk with minimal sample pretreatment; the recoveries were 85–95%. The developed test system is an effective tool for the selective detection of both isomers of OFL, allowing for the avoidance of false negative results. This immunochromatographic approach can be promising for the control of other optically active food toxicants.

## 1. Introduction

Currently, antibiotics are one of the most major contaminants of food products [1,2]. The presence of antibiotic residues in foodstuffs is caused by their intensive use in agriculture for the treatment and prevention of infectious diseases in farm animals and poultry [3]. The consumption of food containing antibiotics is detrimental to the health of not only individuals (provoking gastrointestinal disorders, allergic hypersensitivity, and secondary fungal infections, among other effects), but also poses a significant risk to the population as a whole, as it contributes to the spread of pathogens resistant to various classes of antibiotics [4,5,6]. The inefficiency of antibiotic therapy hinders the treatment of different bacterial infections and may even lead to fatal outcomes. One of the main priorities regarding consumers’ safety is monitoring antibiotics in milk and dairy products, where their residue levels are regulated [7]. Further development in this area could include the detection of antibiotics in the urine and feces of farm animals [8]. The fluoroquinolone (FQ) group of antibiotics has been used in human and veterinary medicine for several decades due to their bactericidal properties (FQs destroy bacteria by inhibiting their vital enzyme DNA gyrase) [9,10,11]. FQs are completely synthetic compounds with high bioavailability, good pharmacokinetic and pharmacodynamic properties, and a broad spectrum of activity against many aerobic Gram-negative and some Gram-positive pathogens. Ofloxacin (OFL) is a second-generation FQ antibiotic, widely used today to treat infectious and inflammatory diseases of the respiratory tract, skin and soft tissues, bones and joints, kidneys, genitals, etc. [12].

The fine structural complementarity of interacting molecules, primarily determined by their spatial organization, is essential for many biological processes [13,14]. To ensure biological functions, the structures of interacting molecules (e.g., receptor and ligand, enzyme and substrate) should have a corresponding steric fit [15]. Many medications are also characterized by a close relationship between their spatial structure and pharmacological effect, i.e., they exhibit stereospecific activity [16,17]. Many synthetic pharmaceuticals have two or more stereoisomers [18]. These include several FQs, in particular, OFL (see its chemical structure in Figure S1a), which is a racemic mixture of levorotatory (S-OFL) and dextrorotatory (R-OFL) enantiomers (pairs of stereoisomers that are mirror images of each other, not superimposable in space) [19]. A *sp*^3^-hybridized carbon atom with four different substituents (asymmetric carbon at the C-3 position on the oxazine ring) is the chiral center of OFL [19]. Typically, only one of the stereoisomers exhibits the required biological activity (eutomer), so the pharmacological activity of racemic drugs is usually associated with the action of only one enantiomer [20]. The second is either not active at all, exhibits less pronounced activity, or even leads to the development of adverse side effects, such as toxicity (distomer). In the case of OFL, its antimicrobial activity is determined by its S-enantiomer (levofloxacin). The antibacterial activity of R-OFL is two orders of magnitude lower than that of S-OFL [19]. Interestingly, the difference in antibacterial activity between the OFL isomers is not associated with various drug transport mechanisms, but rather with varying inhibitory activity against the target enzyme, DNA gyrase [21]. It should be noted that conventional (non-chiral) methods of chemical synthesis produce racemic mixtures, and even the use of stereoselective synthetic approaches does not always result in the production of individual enantiomers. Moreover, the separation of enantiomers is a rather complex, expensive, and labor-intensive process [22]. This leads to manufacturing many synthetic drugs not as individual stereoisomers, but as their mixtures [23,24].

Taking all these factors into account (the widespread use of FQs, including OFL, in veterinary medicine, the possible transit and accumulation of antibiotic residues in food products), and the fact that it is hardly possible to avoid the use of antibiotics in agriculture at present, it is critical to control their content in raw materials and finished food products. The above-mentioned features of the biological action of OFL enantiomers, as well as the specifics of pharmaceutical production, necessitate the control of both optical isomers of OFL (S- and R-), i.e., the enantioselective detection of the antibiotic. Chromatographic methods and capillary electrophoresis, traditionally used for this purpose, along with their well-known benefits (selectivity and sensitivity), have several disadvantages, such as the need for complex equipment, highly qualified personnel, and large resource costs [25,26,27]. This makes these techniques problematic for rapid (often just qualitative) analysis, which is required when a lot of real samples need to be monitored on-site. In this case, immunoanalytical methods based on highly specific antigen–antibody interactions appear to be a promising alternative. These include enzyme-linked immunosorbent assay (ELISA) and immunochromatographic detection (lateral flow immunoassay, LFIA) [28,29,30,31]. Possessing all the advantages of ELISA (sensitivity, specificity, and accuracy), LFIA is a faster, cheaper, and simpler technique. Unlike ELISA, it does not require any specialized equipment (except a conventional scanner and simple software for the quantitative evaluation of the assay results), additional reagents, or specialized operator skills. The analytical tool is an immunochromatographic test strip, which is a multimembrane composite with pre-applied immunoreagents. The analysis involves incubating the test strip with the sample for a short time (10–20 min) and visually assessing the results for the presence/brightness of colored bands resulting from immune interactions.

The production of poly- and monoclonal anti-OFL antibodies and the development of ELISA and LFIA based on them have been reported in several studies, including recent works using labels with new properties and detection tools that allow for high analytical sensitivity (in the nano- and picogram concentration range) in various biological matrices [32,33,34,35,36]. However, the authors generally did not focus on creating test systems that would detect both the eutomer and distomer of OFL but only reported on the production of antibodies with a certain specificity and their use in analyzing the biologically active S-isomer of the antibiotic, with an assessment of the cross-reactivity (CR) of the obtained receptors to the second enantiomer. In another pool of studies, the authors were interested in the mechanisms of enantiospecific recognition of OFL isomers by antibodies without the use of the identified regularities for the development of immunoassays for the detection of an isomer in real samples [37,38] (for more details, see Section 3.5).

In this study, polyclonal antibodies (PAb) for a racemic mixture of OFL enantiomers (rac-OFL) were obtained by rabbit immunization, and conditions for enantiospecific immunochromatographic detection of both isomers with negligible cross-reactivity to the second enantiomer were selected. A double lateral flow test system was developed for the first time that allows for the simultaneous determination of S- and R-OFL using the same PAb and different OFL–protein coating conjugates in a heterologous mode (different haptens were used for PAb production and adsorption on the solid phase). The developed double test system was successfully applied to the determination of the eutomer and distomer in a food matrix (milk). The uniqueness and potential of the obtained results are due to the ability to reveal not only the biologically active OFL isomer but also its less active one, which may be present in medications. The proposed instrument for their selective detection will eliminate false negative results when monitoring agricultural products for contamination with OFL.

## 2. Materials and Methods

### 2.1. Reagents and Materials

In the study, S-OFL from Santa Cruz Biotechnology (Santa Cruz, CA, USA) and R-OFL from MedChemExpress (Monmouth Junction, NJ, USA) were applied. Rac-OFL, gamma-aminobutyric acid (gaba), gold (III) chloride hydrate (HAuCl_4_ × H_2_O), 1-ethyl-3-(3-dimethylaminopropyl) carbodiimide (EDC), *N*,*N*′-dicyclohexylcarbodiimide (DCC), *N*-hydroxysuccinimide (NHS), sodium citrate, bovine serum albumin (BSA), soybean trypsin inhibitor (STI), ovalbumin (OVA), sucrose, tris(hydroxymethyl)aminomethane (Tris), triethylamine, dimethyl sulfoxide (DMSO), and dimethylformamide (DMF) from Sigma-Aldrich (St. Louis, MO, USA) were used. Goat anti-rabbit immunoglobulins (GARI) and donkey anti-goat immunoglobulins (DAGI) were purchased from Arista Biologicals (Allentown, PA, USA). GARI conjugated with horseradish peroxidase (GARI-HRP) were from Jackson Immuno Research Labs (West Grove, PA, USA). A ready-to-use TMB-based substrate solution was obtained from Immunotech (Moscow, Russia). All other chemicals were of analytical grade (Khimmed, Moscow, Russia). All solutions were prepared with ultrapure water with a resistivity of at least 18.2 MΩ × cm (at 25 °C) obtained on a Simplicity^®^ water purification system (Millipore Corporation, Burlington, MA, USA). For ELISA, polystyrene 96-well transparent microplates from Costar (Corning, Tewksbury, MA, USA) were used.

### 2.2. Synthesis of OFL–Protein Conjugates as an Immunogen and Coating Antigens

OFL–protein conjugates were synthesized using carbodiimide activation [39]. As an immunogen, the rac-OFL-–STI conjugate was obtained. For this, rac-OFL (2.5 mg) was dissolved in DMF (0.5 mL), and then, NHS (1.7 mg) and EDC (2.9 mg) were added. The reaction mixture was stirred at room temperature (RT) for 2 h. After that, the activated hapten solution was added dropwise to the STI solution (7 mg) in 50 mM sodium carbonate buffer, pH 9.5 (3 mL), containing triethylamine (50 µL). The resulting reaction mixture was stirred for 2.5 h at RT and then overnight at 4 °C.

Protein derivatives of enantiomers (S-OFL-gaba-OVA and R-OFL-gaba-BSA) were synthesized as coating antigens [40] so that the protein fragment of the conjugates differed from that of the immunogen. Gaba was used as a linker between the hapten and the protein, which was intended to guarantee the best spatial presentation of the hapten for interaction with PAb. For this, S-OFL or R-OFL (3 mg each), NHS (2.3 mg), and DCC (2.6 mg) were diluted in DMF (1 mL) and stirred overnight at RT. After that, gaba linker (2.2 mg) was added to the activated solution and incubated in the same conditions. Either OVA or BSA was diluted in the 50 mM sodium carbonate buffer, pH 9.5 (5 mL), and then, DMF (200 µL) was added dropwise to both protein solutions. After that, activated hapten-gaba solutions were added dropwise to the OVA and BSA solutions, and the reaction mixtures were mixed for 5 h at RT. The resultant reaction mixtures were centrifuged at 5000× *g* for 10 min to remove the formed precipitate.

All conjugates were purified by dialysis against 50 mM phosphate-buffered saline containing 100 mM NaCl, pH 7.4 (PBS). The UV-vis spectra of the rac-OFL-STI, S-OFL-gaba-OVA, and R-OFL-gaba-BSA conjugates were recorded using a Libra UV-vis spectrophotometer (Biochrom, Cambridge, UK). The conjugates were divided into aliquots and stored at −18 °C.

### 2.3. Production of PAb

The maintenance and experiments with animals were carried out in accordance with Directive 2010/63/EU on the protection of animals used for scientific purposes. Immunization of a rabbit was carried out in accordance with the legislation of the Russian Federation and was approved by the Local Ethics Committee at the Research Center of Biotechnology of the Russian Academy of Sciences, and the investigation was performed according to the ethical guidelines (protocol 09 dated 25 October 2024). To raise anti-OFL PAb, the immunization scheme described in [41] was applied, which demonstrated its effectiveness for the generation of PAb to low-molecular-weight antigens. A Chinchilla rabbit weighing 3.5 kg was immunized with rac-OFL-STI conjugate at day 1 (subcutaneously, with complete Freund’s adjuvant, 1:1 *v*/*v*, 1 mg/rabbit by protein) and at days 22, 36, 50, and 64 (subcutaneously, with incomplete Freund’s adjuvant, 1:1 *v*/*v*, 0.5 mg/rabbit by protein). At days 44, 57, and 72, blood was collected from the ear vein. The antiserum was separated (hereafter called AS1, AS2, and AS3), divided into aliquots, and stored at −20 °C.

### 2.4. Antisera Testing by ELISA

S-OFL-gaba-OVA or R-OFL-gaba-BSA conjugates (0.9 µg/mL, 100 µL in PBS) were immobilized in the microplate wells overnight at 4 °C. Then, the microplate was washed four times with PBS containing 0.05% Triton X-100 (PBST), and AS1–3 in dilutions from 1:100 to 5.9 × 10^6^ (100 µL in PBST) were added to the wells and incubated for 1 h at 37 °C. After washing the microplate as described above, GARI-HRP (1:5000 dilution, 100 µL in PBST) was added to the wells and incubated for 1 h at 37 °C. After washing, the HRP activity was registered. To do this, a TMB substrate solution (100 µL) was added to the microplate wells and incubated for 15 min at RT. The reaction was stopped by adding 1 M sulfuric acid (50 µL), and the optical density (OD) was measured at 450 nm (OD_450_) on a Zenyth 3100 microplate spectrophotometer (Anthos Labtec Instruments, Wals, Austria). Based on the obtained dependencies, PAb titers were calculated.

For the indirect competitive ELISA (icELISA), S-OFL-gaba-OVA or R-OFL-gaba-BSA conjugates were immobilized as described above. Next, solutions of S-OFL or R-OFL (1 µg/mL–0.1 pg/mL, 50 µL in PBST) and AS3 (in dilutions of 1:2000 and 1:5000 for S-OFL and R-OFL detection, respectively, 50 µL in PBST) were added to the wells and incubated for 1 h at 37 °C. Then, successive stages of incubation with GARI-HRP and detection of the formed immune complexes were carried out as described above. Finally, the analytical performance of the icELISA was assessed (see Section 2.10).

### 2.5. Synthesis of Gold Label and Its Conjugation with Antibodies

Gold nanoparticles (AuNPs) were synthesized by the citrate technique [42]. To perform this, 1.0 mL of 1% HAuCl_4_ solution was added to 97.25 mL of deionized water and heated to boiling. After that, 1.75 mL of 1% sodium citrate solution was added and stirred while boiling for another 20 min. The synthesized AuNPs (OD_520_ = 1) were stored at 4 °C for at least 1 month.

AuNPs were conjugated with GARI following [43]. Before complexation, the pH of AuNPs was adjusted to 9.2 with 0.1 M K_2_CO_3_, and GARI were dialyzed against 10 mM Tris-HCl, pH 9.0. GARI (6.2 μg/mL) were added to AuNPs and incubated for 2 h at RT under stirring. Next, a 10% BSA solution was added (1:40, vol./vol.) and stirred under the same conditions for 20 min. The synthesized GARI–AuNPs conjugate was centrifuged at 12,000× *g* for 20 min, and the residue was redispersed in 2.0 mM of Tris buffer, pH 7.5, containing 0.25% BSA, 0.25% Tween-20, and 1% sucrose to obtain a concentrated preparation (OD_520_ = 28). The GARI–AuNPs conjugate was stored at 4 °C.

To characterize the dimensional parameters and morphology of the label, transmission electron microscopy (TEM) and dynamic light scattering (DLS) measurements were implemented on a JEM-100C microscope (JEOL, Tokyo, Japan) and a Zetasizer Nano ZS 90 (Malvern, UK), respectively. UV–vis spectra were registered using a Libra S50PC spectrophotometer (Biochrom, Cambridge, UK).

### 2.6. Manufacturing of Test Strips

Test strips were obtained using a nitrocellulose working membrane (CNPCSS12-L2-H50 with 15 µm pore size) fixed on a plastic support and an AP045 adsorption pad (both from Advanced Microdevices, Ambala Cantt, India). For immobilization of the reagents, an Iso-Flow dispenser (Imagene Technology, Hanover, NH, USA) was used with a loading rate of 0.1 µL/mm. As an immobilization buffer, PBS was used.

For the individual LFIAs, two zones—a test zone (TZ) and a control zone (CZ)—were formed on the working membrane. TZ was obtained by the application of S-OFL-gaba-OVA or R-OFL-gaba-BSA conjugate (0.5 mg/mL) for S-OFL and R-OFL detection, respectively. In the CZ, DAGI (0.05 mg/mL) were absorbed in both LFIAs. The working membranes with applied reagents were dried overnight at RT and for 2 h at 37 °C. Then, the adsorption pad was fixed on the plastic support, the bottom of the plastic support was cut off, and the obtained multicomposites were slit into test strips of 2.9 mm width with a guillotine (KinBio, Shanghai, China).

For the double LFIA, three zones—two TZs (TZ1 and TZ2) and a CZ—were formed. TZ1 was formed by the application of R-OFL-gaba-BSA conjugate (0.5 mg/mL). In TZ2, S-OFL-gaba-OVA (0.75 mg/mL) was adsorbed. The CZ was obtained as described in the individual test systems. All other manipulations were performed as described above. Upon storage of the test strips in sealed bags with silica gel at RT, the analytical performance of the test systems did not change for at least 2 months.

### 2.7. Sample Preparation Before LFIA and Obtaining Spiked Samples

Cow milk with 2.5% fat content, certified as being free of antibiotics and pesticides, was purchased from an organic food store. For sample preparation, a 5-fold dilution by PBS containing 1% Tween-20 (PBS_TW1_) was implemented. Four spiked samples were prepared using concentrations of 0.56 and 1.7 ng/mL (for S-OFL) and 4.1 and 12.3 ng/mL (for R-OFL), containing both isomers and possible combinations.

### 2.8. Individual LFIAs of S-OFL and R-OFL

S-OFL or R-OFL standard solutions (1000–0.01 ng/mL, 20 μL in PBS_TW1_) and AS3 (in 1:25 dilution, 20 µL in PBS_TW1_) were mixed and incubated for 3 min. Then, test strips were immersed in the mixtures, incubated for 5 min, and taken out. Next, test strips were washed by immersing in PBS_TW1_ (50 μL) for 3 min. After that, test strips were taken out, put horizontally, and 5.0-μL aliquots of GARI-AuNPs (with a dilution corresponding to the OD_520_ = 4.0) were applied close to the bottom of the working membrane and kept for 1 min. After washing the test strips for 5 min, they were taken out, blotted, and scanned using a CanoScan LiDE 90 scanner (Canon, Tokyo, Japan). The coloration intensity of bands was evaluated by the TotalLab TL120 v2009 software (Nonlinear Dynamics, Newcastle upon Tyne, Great Britain). The assay analytical parameters were calculated as described in Section 2.10.

The CR was assessed following [44,45] as *IC*_50target antigen_/IC_50cross-reactant_ × 100%, where *IC*_50_s were inflection points of the corresponding calibration curves.

### 2.9. Double LFIA

S-OFL and R-OFL standard solutions (333-0.006 ng/mL for S-OFL and 417-0.007 ng/mL for R-OFL, 10 μL in PBS_TW1_ both) and AS3 (in 1:60 dilutions, 20 µL in PBS_TW1_) were mixed to prepare the following pairs of S-OFL/R-OFL concentrations: 417/333, 139/111, 46/31, 11/12, 5.1/4.1, 1.7/1.4, 0.6/0.5, 0.2/0.15, 0.06/0.05, 0.02/0.017, 0.007/0.006, and 0.0008/0.002 ng/mL. The obtained mixtures were incubated for 3 min. All other steps were the same as for individual test systems (see Section 2.8), including the processing of the colored bands.

### 2.10. Evaluation of the Immunoassay Results and Statistics

Dependencies of OD_450_ (for the icELISA) or TZ coloration (for the LFIA) (*y*) versus OFL concentrations (*x*) were built and processed by a four-parameter logistic function using OriginPro 9.0 program (OriginLab, Northampton, MA, USA). The instrumental LODs (both in the icELISA and LFIA) were calculated using the Three Sigma approach [46]. As the working range of the detectable concentrations (WR), a 20–80% signal decrease from its maximal value was considered. The cutoff (or visual LOD) of the LFIA was estimated as the analyte concentration causing the full disappearance of TZ coloration, which corresponded to a colorimetric signal of less than 600–700 relative units (RU) of the colorimetric signal. All quantitative experiments were made in triplicate, and the means ± SE (standard errors) of OFL concentrations were assessed. To assess the assay reproducibility and repeatability, intra-assay’s and inter-assay variation coefficients were assessed (n = 6). For the milk sample analysis, five repeats were performed.

## 3. Results and Discussion

### 3.1. Obtaining Key LFIA Components and Their Characterization

#### 3.1.1. Production and Testing of PAb

One of the critical objectives of this study, necessary for the successful development of the test system, was the production of receptors for target analytes, enabling the differential detection of the S-OFL and R-OFL enantiomers. For this purpose, a racemic mixture, containing both S- and R-isomers, was used as a hapten in the conjugate for immunization. The spectrophotometric characterization of hapten–protein conjugates synthesized as an immunogen and coating antigens confirmed the successful modification of the protein with haptens, presenting peaks characteristic of the protein (at 280 nm) and OFL (at 292 and 332 nm) (Figure S1b). As a result of immunization, three antisera (AS1–3) were obtained, which were analyzed by titers defined as the highest dilution of antiserum that ensures the reliable detection of the interaction with the immobilized antigen. The PAb titers ranged from 1:20,000 to 1:500,000.

**Figure 1:**
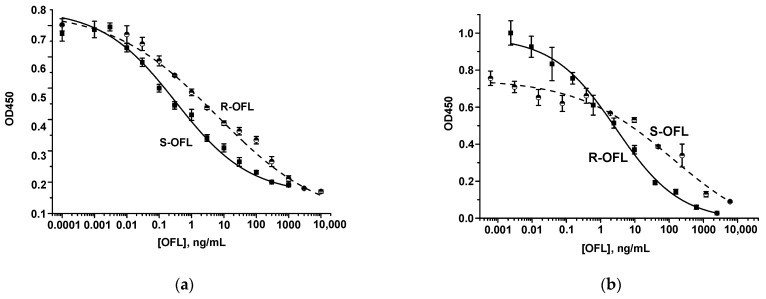
Calibration curves of S-OFL and R-OFL in the icELISAs using S-OFL-gaba-OVA/AS3 (**a**) and R-OFL-gaba-BSA/AS3 (**b**) combinations.

The icELISA, based on the competitive interaction of free analyzed and immobilized antigens with specific antibodies, was applied to select the antiserum providing the most sensitive determination of the antigens. It was important that the test system configuration could ensure separate enantiospecific determination of OFL isomers, i.e., CR of PAb with the second isomer would be absent or negligible (~5–7%) under proper assay conditions. Antisera from all three bleedings were preliminarily tested in an icELISA with immobilization of coating antigens, in which a hapten component was the same as the detected antigen (Figure S2). The antiserum isolated as a result of the third bleeding (AS3), which provided the best enantiomer distinguishing in the icELISA, was selected as optimal for the simultaneous detection of S- and R-isomers. Calibration curves of OFL isomers in the selected coating conjugate/target analyte/second isomer/antiserum conditions (R-OFL-gaba-BSA/R-OFL/S-OFL/AS3 and S-OFL-gaba-OVA/S-OFL/R-OFL/AS3) are shown in Figure 1.

LODs, WRs, and CR values are presented in Table 1. The above combinations of immunoreagents were further used to develop the LFIAs.

The CRs of the produced PAb with a panel of FQs including difloxacin, enoxacin, ciprofloxacin, orbifloxacin, enrofloxacin (ENR), garenofloxacin (GAR), lomefloxacin, flumequine, sparfloxacin, marbofloxacin (MAR), pipemidic acid, nadifloxacin, tosufloxacin, rufloxacin (RUF), S-OFL, rac-OFL, cinoxacin (CIN), moxifloxacin, danoloxacin, pefloxacin (PEF), clinafloxacin, pazufloxacin, oxolinic acid, and sarafloxacin (all from Sigma-Aldrich, St. Louis, MO, USA) were also tested. It was shown that anti-rac-OFL-STI PAb noticeably cross-reacted with S-OFL (83.3%), RUF (26.9%), MAR (12.1%), and GAR (7.7%). For PEF, ENR, and CIN, the CRs were lower (1.8, 1.1, and 0.77%) (Figure S3). For all other structural analogs, the CRs were <0.01%.

#### 3.1.2. Obtaining, Characterization, and Conjugation of AuNPs

**Figure 2:**
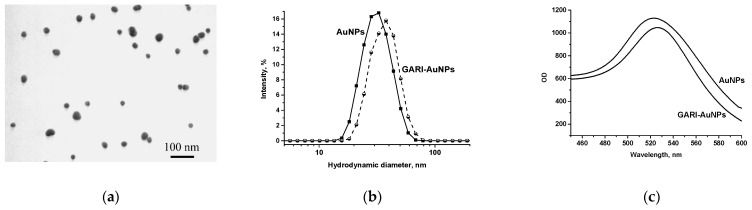
Characterization of AuNPs and GARI–AuNPs by TEM (for AuNPs) (**a**) and DLS (**b**) measurements and UV-vis spectrophotometry (**c**).

AuNPs, which have been proven to be an effective immunochromatographic marker in analyses of various compounds, were synthesized [47,48]. AuNPs are a stable marker obtained by a simple and reproducible method of gold salt reduction with citrate. It is well conjugated with biopolymers and provides bright red bands on test strips as a result of immune interactions. The AuNPs obtained in this work were characterized by TEM and DLS measurements and UV-vis spectroscopy (Figure 2). According to TEM, the AuNPs’ diameters were 26.7 ± 3.6 nm, and their ellipticity was 1.3 ± 0.1 (in total, 96 nanoparticles were processed) (Figure 2a). DLS data revealed that the AuNPs hydrodynamic diameter was 32.7 nm with a Pdi of 0.192 (Figure 2b). The difference in the estimated TEM- and DLS-based dimensions is explained by the hydration shell contribution, which is considered in the DLS measurements. As can be seen from the spectrum, the absorption maximum was observed at a wavelength of 523 nm (Figure 2c), which, in accordance with the previously established relationship between the average diameter and the peak of the absorption spectrum of spherical AuNPs [49], corresponds to a diameter of 28.5 nm.

The label was conjugated with anti-species antibodies specific to rabbit immunoglobulins (GARI) for subsequent implementation of the LFIA in an indirect competitive mode. The principle of this format is similar to that of the icELISA, and the GARI-AuNP conjugate is used to detect immune complexes formed on the test strip. For complexation, non-covalent immobilization of the protein on the surface of the AuNPs was performed using GARI at a concentration ensuring the formation of a protein monolayer (6.2 µg/mL) [49]. The diameter of the conjugate according to the UV-vis spectrum was 34 nm (Figure 2c), thus increasing by 5.5 nm compared to that of the initial AuNPs due to the formation of a protein cover on their surface. The hydrodynamic diameter of the conjugate was 37.8 nm with a Pdi of 0.197, according to DLS data, confirming the conjugate’s formation (Figure 2b).

### 3.2. Individual LFIAs OFL Enantiomers

Before creating the double LFIA, individual test systems were developed for the separate determination of OFL enantiomers using a combination of immunoreagents selected in the icELISA. Accordingly, S-OFL-gaba-OVA or R-OFL-gaba-BSA conjugates were applied to the TZ to detect S-OFL and R-OFL, respectively. The CZ was formed by immobilizing anti-species antibodies (DAGI) in both cases. In the competitive LFIA (typically used for low-molecular-weight analytes), if the target analyte is absent in the test sample, specific antibodies interact with anti-species-labeled antibodies, and this complex binds to the immobilized analyte in the TZ, forming a colored line. Excess labeled antibodies are concentrated in the CZ, which is followed by CZ coloration. Consequently, in the absence of the analyte, two red lines are visualized on the test strip. If the analyte is present in the sample, it inhibits the interaction of specific antibodies with the immobilized antigen in the TZ, reducing TZ color intensity proportionally to the antigen concentration until the band completely disappears. Accordingly, an inversely proportional relationship between OFL concentration and colorimetric signal intensity is observed.

To ensure a minimal LOD and high colorimetric signals, the test systems were optimized, including the selection of appropriate concentrations of free and bound reagents, the sequence and duration of assay steps, the reaction medium composition, etc. A balance had to be maintained between the receptor concentration (to increase the sensitivity of competitive detection, it is usually required to be reduced) and ensuring a reproducible signal and, consequently, the accuracy of the assay. The development of immunanalytical test systems based on PAb may possess some difficulties due to the presence of a pool of antibodies with varying specificities and other constituents of a multicomponent polyclonal antiserum. We took into account the possible growth of a background signal at the zero point (when testing samples containing no PAb or analyte) or/and the absence of a signal, which is expected as a result of immune interactions associated with nonspecific reactions of serum components with the immunoreagents of the test system. These effects can be compensated for by employing various approaches, i.e., pre-treatment of the working membrane, addition of blocking agents to the reaction medium, washing the membrane after the analysis, etc. [50,51].

In this study, to prevent nonspecific interactions, the number of membrane carriers was reduced to the necessary minimum, namely, two. The test strip was composed of the working membrane, on which the immune reactions occurred, and the top absorbent pad, which soaked up the fluid after all interactions. Accordingly, the plastic support was cut to the lower edge of the working membrane, thereby reducing the total volume of the test sample to 40 µL (as opposed to the 100 µL required for a full-size test) and shortening the duration of complete sample absorption. The immune interaction was carried out in a separate vessel (in a microplate well) (stage 1), into which the test strip was then immersed and incubated (stage 2). After incubation, the test strip was removed, and the labeled anti-species antibodies were applied and incubated again (stage 3). Interactions were carried out in a medium with a high detergent content (1% Tween-20, PBS_TW1_) to minimize nonspecific effects. Stages 2 and 3 were accompanied by washing the test strip in the same medium. Without washing after stage 2, it was not possible to adequately visualize TZ coloration (Figure S4). After stage 3, washing was necessary to allow the passage of GARI-AuNPs with the liquid flow through the membrane carriers. Upon the addition of GARI-AuNPs to the reaction mixture during stage 1, an undesirable background coloration appeared on the TZ, possibly due to the use of a complex biological matrix (antiserum), whose components promoted nonspecific interactions on the membrane. To avoid this, we carried out the LFIA in a sequential format with a washing step between stages. It should be noted that the preliminary isolation of the immunoglobulin fraction from the serum may reduce the nonspecific interactions caused by the whole antiserum. This may be used in the future design of the test, allowing for a reduction in the number of stages and the acceleration of the overall analytical procedure.

**Figure 3:**
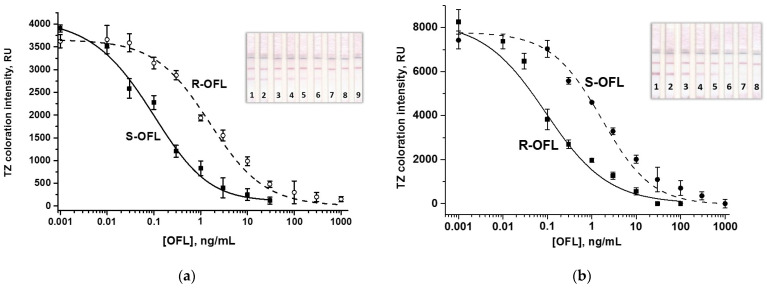
Calibration curves of S-OFL (**a**) and R-OFL (**b**) and the corresponding test strips in the individual LFIAs. By the dashed lines, calibration curves of the non-target OFL isomers are shown. Numbers on the bottom of test strips indicate OFL concentrations of 0 (1), 0.01 (2), 0.03 (3), 0.1 (4), 0.3 (5), 1 (6), 3 (7), 10 (8), and 30 ng/mL (9)—for S-OFL LFIA (**a**) and 0 (1), 0.03 (2), 0.1 (3), 0.3 (4), 1 (5), 10 (6), 30 (7), and 100 ng/mL (8) for R-OFL LFIA (**b**). For the blank samples (with no analyte), colorimetric signals correspond to 3906 ± 77 RU and 8263 ± 558 RU for S-OFL (**a**) and R-OFL (**b**) detections, respectively.

Optimization also included varying the concentration of the immobilized conjugate (Figure S5) and DAGI in the antiserum dilution (Figure S6), the amount of added antibody-labeled conjugate, and the duration of the assay steps (see varied and selected LFIA conditions in Table S1). Despite the multi-stage analysis, the overall testing time was short: stage 1 took 3 min, stage 2 proceeded within 5 min, followed by a 3 min washing, and stage 3 took 1 min, followed by a 5 min washing (Table S1). Increasing the duration of the assay steps did not affect the analytical performance of the test system. Thus, the total LFIA time was 15 min, fully satisfying the requirement for rapid tests. The calibration curves of the analytes after all optimizations and the appearance of the test strips are shown in Figure 3.

Despite the results of the icELISA, the conditions of which guaranteed the recognition of each OFL enantiomer, a control for CR with the second enantiomer was carried out for both immunochromatographic test systems. According to Figure 3, CRs of PAb with the non-target enantiomer were negligible, similar to the corresponding icELISAs (see Section 3.1). In the monoparametric LFIAs, LODs/cutoffs/WRs/CRs were 0.004/3/0.01-0.7/5.2 and 0.003/10/0.01-0.84/5.5 (ng/mL/ng/mL/ng/mL/%) in the S-OFL and R-OFL test systems, respectively.

### 3.3. Double LFIA for Simultaneous Detection of S-OFL and R-OFL

**Figure 4:**
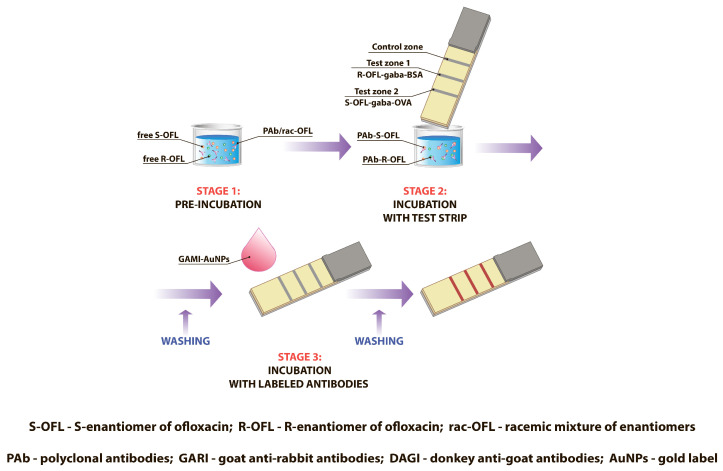
Scheme of the simultaneous determination of S-OFL and R-OFL in the double LFIA.

A multiparametric test system combines components of several monoparametric tests, meaning the number of TZs corresponds to the number of detected analytes. In our case, three analytical zones were formed on the test strip: two TZs and one CZ. Accordingly, if the sample contains one or two analytes, inhibition of the colorimetric signal will be observed in the corresponding TZs (see the double LFIA scheme in Figure 4).

**Figure 5:**
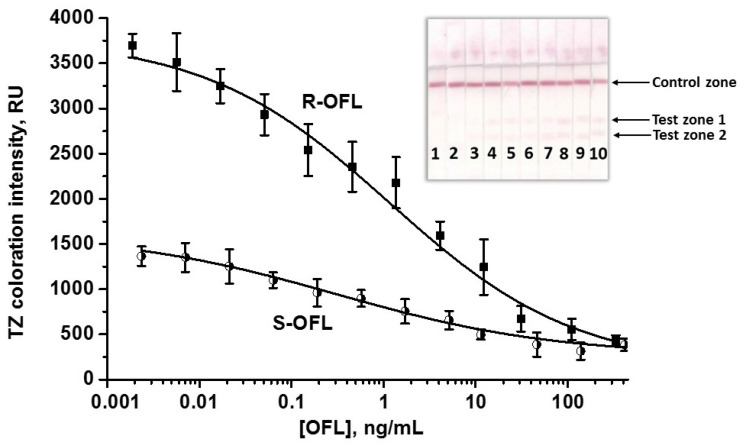
Calibration curves of S-OFL and R-OFL in the double LFIA and images of the test strips. Numbers on the bottom of test strips indicate OFL concentrations of 139 (1), 46 (2), 11 (3), 5.1 (4), 1.7 (5), 0.6 (6), 0.2 (7), 0.06 (8), 0.02 (9), and 0.007 (10) ng/mL—for S-OFL LFIA (**a**) and 111 (1), 31 (2), 12 (3), 4.1 (4), 1.4 (5), 0.45 (6), 0.15 (7), 0.05 (8), 0.02 (9), and 0.006 (10) ng/mL for R-OFL LFIA (**b**). For the blank sample (with no analytes), colorimetric signals correspond to 1365 ± 107 RU and 3696 ± 130 RU for S-OFL and R-OFL detections, respectively.

It is known that TZs’ locations on the test strip can affect the analytical parameters of the multiparametric test system [52,53]. Therefore, the development of a double LFIA involves optimization, which includes, among other things, the selection of TZs arrangement. In our case, the following locations were tested (top-down): CZ → TZ1 (S-OFL-gaba-OVA) → TZ2 (R-OFL-gaba-BSA) (1st variant) and CZ → TZ1 (R-OFL-gaba-BSA) → TZ2 (S-OFL-gaba-OVA) (2nd variant, see the variable parameters of the test system in Table S1). It was shown that the 2nd variant (when S-OFL-gaba-OVA is below) is optimal, providing a higher colorimetric signal in TZ2 (Figure S7). This may be due to the higher affinity of AS3 toward the R-isomer, so when the R-OFL TZ is positioned below (the 1st variant), most of the antibodies moving upward with the fluid flow will readily interact with the immobilized R-OFL-gaba-BSA conjugate in TZ2. The remaining antibodies will interact with S-OFL-gaba-OVA in the TZ1. When the S-OFL-gaba-OVA conjugate is immobilized below in TZ2, the effect is opposite: PAb that remained free after step 1 will primarily bind to this conjugate, providing the formation of a brighter band (Figure S7). To further ensure the brightness of TZ2, the S-OFL-gaba-OVA conjugate was adsorbed at a higher concentration compared to the individual test—0.75 mg/mL (Table S1). This allowed the use of the specific AS3 antiserum at a slightly higher dilution than in individual tests (1:60, see the results of the antiserum dilution selection in Figure S8), maintaining the reproducibility of the results and ensuring a low LOD for both isomers. The volume of the applied GARI-AuNPs aliquot (5 µL), the concentration of R-OFL-gaba-BSA in TZ1 (0.5 mg/mL) and DAGI in CZ (0.05 mg/mL), as well as the duration of the assay steps (3 min (1) → 5 min (2) → 3 min (washing) → 1 min (3) → 3 min (washing)) were the same as in the individual tests. The maxima of the analytical signals were lower than those registered in monoparametric test systems, because specific PAb (as well as labeled GARI), even added at lower concentrations than in individual tests, were consumed in the binding with both analytes. However, this allowed the assay to maintain a good analytical performance and even decreased the LOD of S-OFL down to 0.001 ng/mL. To make the signals comparable in the monoparametric and double LFIAs, the antibody concentration has to be increased; however, in this case, the assay sensitivity would suffer. Hence, a high sensitivity was opted for to maintain the reproducibility of the analytical signals. The calibration curves and the appearance of the test strips after double LFIA are presented in Figure 5. In the double LFIA, the LODs/cutoffs/WRs were 0.001/10/0.006-0.14 and 0.007/30/0.05-29 ng/mL for S-OFL and R-OFL detection, respectively. As can be seen, the cutoffs of the analytes in the double test system have slightly increased. However, this corresponds to the idea about possible changes in the parameters of multiparametric LFIA compared to those of the related individual test systems [52,53] and allows the detection of OFL with high sensitivity.

### 3.4. Application of the Double LFIA for OFL Detection in Milk

Potential practical application of the developed double LFIA for the simultaneous detection of the OFL eutomer and distomer requires testing it on real samples. Milk was used as a real matrix as a foodstuff that may be contaminated with antibiotics used in agriculture for farm animal therapy [7]. A sample of 2.5% fat milk was purchased from a local organic food supermarket. In the case of testing liquid samples, LFIA can sometimes be applied without preliminary sample preparation [54,55]. However, for a multicomponent milk matrix, which contains proteins, lipids, and lactose, and can affect the testing process and its results, some pretreatment is often required. In this study, a sample preparation that included dilution with a buffer (PBS_TW1_) was proposed. An analysis of the undiluted milk yielded poor results: milk matrix components clogged the pores of the working membrane, resulting in nonspecific coloration of the entire membrane, making immune interactions in the TZs undetectable (Figure S9). The same was true for a 2-fold dilution. The WRs of S-OFL and R-OFL in 5- and 10-fold diluted milk were comparable. Given the requirement for minimal matrix dilution for sensitive testing, a 10-fold dilution was excessive, and 5-fold diluted milk was selected as a pretreated sample (Table S2). For recovery assessment, the initial milk sample was spiked with S-OFL and R-OFL, then diluted as required, and analyzed in a double LFIA. Spiking concentrations were selected from the WRs, namely, 0.56 and 1.7 ng/g for S-OFL and 4.1 and 12.3 ng/g for R-OFL. Samples containing isomers in five-fold concentrations were prepared in all combinations of S-OFL/R-OFL and diluted by PBS_TW1_ to obtain the following pairs: 0.56/0.41 ng/g, 0.56/12.3 ng/g, 1.7/4.1 ng/g, and 1.7/12.3 ng/g. Images of the test strips after the LFIA in milk samples are presented in Figure S10. The obtained recovery values are presented in Table 2.

As can be seen, the double LFIA allows for the effective detection of both enantiomers of the antibiotic in milk (recoveries were 85–95%), regardless of the selected combinations of concentrations. To validate the LFIA results, the icELISA was carried out as a reference method. The same milk samples, spiked with S-OFL and R-OFL, were tested by the icELISA, and the recoveries for each concentration were evaluated and compared with those assessed by the double LFIA. The good comparability of the obtained results (Table 2) confirms the accuracy and reliability of the developed approach and allows us to recommend it for the sensitive and rapid detection of the OFL eutomer and distomer in food matrices.

### 3.5. Novelty and Originality of the Study

It was interesting to compare the results obtained in this study with those described in the literature. The search was conducted using several queries. First, we sought studies on the development of immunoassays for the differential detection of isomers of biologically active compounds using antibodies of given specificity. The search for such studies in the area of LFIA was of particular interest. Second, enantiospecific LFIAs of food contaminants, particularly antibiotics, were looked for. Third, studies on the creation of multiparametric test systems for the simultaneous, rather than independent, determination of enantiomers were of special interest. The literature investigation revealed that more or less successful attempts to produce stereospecific antibodies and implement stereospecific immunoassays have been made. Thus, it was determined that the production of chiral monoclonal antibodies (MAb) to the β-agonist salbutamol (SAL) [56], enantiospecific PAb and recombinant antibodies to the herbicide metolachlor [57,58], PAb to the ectoparasiticide bioallethrin [59], MAb and their Fab-fragments to the antibiotic gatifloxacin [51,60], and MAb to chloramphenicol [61] (all these are relevant food contaminants) have been described. The obtaining of PAb specific to styrene, MAb and their Fab-fragments against the anesthetic ketamine, MAb to medications selegiline and oxaprotiline, as well as against some other compounds characterized by stereoisomerism, were also reported. In most of the found studies, stereospecific determination of analytes was carried out by ELISA.

Enantiospecific MAb and PAb to OFL have also been prepared [33,37,38]. In two of these works, the authors’ tasks included understanding the principles of stereospecific recognition of chiral haptens by antibodies [37] and application of molecular modeling for hapten epitope prediction [38]. The ELISA was applied as an auxiliary way to assess the specificity of the obtained receptors, and not as an analytical tool for detecting target analytes. According to reference [37], the specificity of antibodies was directly determined by the type of hapten in the immunogen used. Immunization of animals with S-OFL and R-OFL as haptens led to the production of PAb with CRs to S-/R-OFL of 100/21.3% and 6.6/100%, respectively, in a homologous ELISA (when the same hapten is used as an immunogen and a coating antigen). Immunization by the racemic mixture resulted in 100% CRs of PAbs to S-, R-, and rac-OFL. In [38], the specificity of the anti-S-OFL and anti-R-OFL MAb to the target OFL enantiomer was studied using ELISA, and molecular modeling methods were used to predict the structures of OFL epitopes. In a homologous ELISA, anti-S-OFL MAb exhibited CRs of 100/18.8/90% to S-OFL/R-OFL/rac-OFL, respectively, and anti-R-OFL MAb showed CRs of 8.6/100/56.4% for the same analytes. So, individual stereospecific determination was possible. A heterologous ELISA (with immunizing and competitor haptens of different molecular structures) showed CRs of 6-100% depending on the coating antigen–MAb pair and therefore, also allowed detection of the analyte of interest in the chosen conditions [38].

In the study [33], PAb to S-OFL and rac-OFL haptens were produced. As in previous investigations, ELISA was used to study the CRs of PAb to isomers and a racemate. It was demonstrated that maximum specificity is achieved when using PAb against S-OFL when implementing a homologous ELISA. In this case, CRs to S-, R-, and a racemic mixture were 100, 8, and 30%, respectively. PAb against rac-OFL had high CRs with isomers and racemate (20–250%), both in homologous and heterologous ELISAs. Therefore, only anti-S-OFL PAb could be used for the specific analysis of exactly S-OFL, and no receptors were available to detect R-OFL or effectively distinguish them if necessary. Hence, the anti-S-OFL PAb were used to develop monoparametric LFIA of S-OFL with CR to R-OFL of 12% [33].

The second study on the development of monoparametric stereospecific immunochromatographic analysis is a study in which magnetic-assisted LFIA based on chiral MAb against the R-isomer of SAL was created [56]. There, the R-SAL was used to produce MAb, and a homologous LFIA was carried out. The CRs for R-SAL, S-SAL, and rac-SAL were 100, 1.9, and 80%, respectively. Thus, as in the previous case, the developed test system allowed the determination of only one isomer and did not offer a universal receptor and/or analysis conditions that would allow for the determination of both isomers with high specificity.

Therefore, to our knowledge, multiparametric immunochromatographic test systems for the simultaneous determination of the eutomer and distomer of any compound do not currently exist. Thus, this study is a unique development in which the same receptor was employed for the simultaneous stereospecific detection of two enantiomers. As such a universal receptor, the PAb produced against rac-OFL as a hapten were used in a the heterologous LFIA to simultaneously determine S-OFL and R-OFL and distinguish them using corresponding coating antigens (S-OFL-protein and R-OFL-protein conjugates for S-and R-isomers detection, respectively) with negligible CR to each other (~5%). The possibility of the successful application of such a test system for the rapid and sensitive determination of OFL isomers in the food matrix emphasizes its importance and significance from a practical point of view.

## 4. Conclusions

Overall, the first simultaneous immunochromatographic detection of levorotatory and dextrorotatory enantiomers of antibiotic OFL has been developed. The stereospecific recognition of S- and R-enantiomers using the same polyclonal antiserum was made possible by, firstly, using a racemic mixture of isomers (i.e., S-OFL and R-OFL) as a hapten for immunogen synthesis, due to which the pool of the produced polyclonal antibodies contained immunoglobulins specific to both isomers. Secondly, for their effective differentiation, we used the corresponding coating antigens, namely, S-OFL-gaba-OVA and R-OFL-gaba-BSA for S-OFL and R-OFL, respectively. The accurate selection of the assay conditions allowed for the possibility of the isomers’ differentiation in the corresponding zones of the test strip using the same polyclonal antiserum, which was a feature of this work. High sensitivity (LODs/cutoffs of S-OFL and R-OFL were 0.001/10 and 0.007/30 ng/mL, respectively) was achieved, and the rapidity of testing (total assay duration was 15 min) was fulfilled. The suitability of the created test system for the simultaneous determination of S- and R-isomers of OFL in the milk matrix with minimal sample preparation was proven. The developed approach is promising when the control of several stereoisomers of the same synthetic compound is required in potentially contaminated food or biological matrices.

## Figures and Tables

**Table 1:** Analytical characteristics of icELISAs of S-OFL and R-OFL.

Detected Analyte	LOD, ng/mL	WR, ng/mL	CR with the Second Enantiomer, %
S-OFL	0.001	0.009–12	5.2
R-OFL	0.03	0.14–55	2.1

**Table 2:** Recoveries of S-OFL and R-OFL in the double LFIA and icELISA (as a reference method) of milk samples.

Immunoassay Mode								
	Double LFIA				icELISA			
OFL	S/R	S/R	S/R	S/R	S	S	R	R
Added, ng/g	0.56/12.3	1.7/12.3	0.56/4.1	1.7/4.1	0.56	1.7	4.1	12.3
Revealed, ng/g	0.52 ± 0.04/ 11.0 ± 0.81	1.54 ± 0.2/ 11.1 ± 1.2	0.49 ± 0.06/ 3.6 ± 0.4	1.61 ± 0.13/ 3.5 ± 0.4	0.60 ± 0.02	1.85 ± 0.04	3.9 ± 0.1	12.5 ± 0.1
Recovery ± SD *, %	92.2 ± 7.5/ 89.8 ± 6.6	90.7 ± 10.9/ 90.3 ± 10.3	87.2 ± 3.7/ 87.0 ± 10.0	94.9 ± 8.2/ 84.7 ± 8.7	107.1 ± 2.9	104.8 ±1.9	95.1 ± 2.6	105.6 ± 1.1

* standard deviation.

## Data Availability

The original contributions presented in the study are included in the article and Supplementary Materials; further inquiries can be directed to the corresponding author.

## Supplementary Materials

The following supporting information can be downloaded at: https://www.mdpi.com/article/10.3390/bios15120765/s1. Figure S1: Structure of the OFL molecule (from https://pubchem.ncbi.nlm.nih.gov) (a) and UV-vis spectra of S-OFL (1), R-OFL (2), BSA (3), OVA (4), rac-OFL-STI (5), S-OFL-gaba-OVA (b), R-OFL-gaba-BSA (7), and STI (8) (b); Figure S2: Calibration curves of S-OFL and R-OFL in the icELISA carried out for pre-selection of antiserum for further studies using AS1 (a,d), AS2 (b,e), and AS3 (c,f) and S-OFL-gaba-OVA (a–c) and R-OFL-gaba-BSA (d–f) immobilized conjugates; Figure S3: Calibration curves of (fluoro)quinolones cross-reacted with anti-rac-OFL-STI PAb in the ELISA; Figure S4: Images of test strips without washing after stage 2 (on the example of monoparametric LFIA of S-OFL). Numbers at the bottom of test strips correspond to S-OFL concentrations of 0 (1), 0.01 (2), 0.03 (3), 0.1 (4), 0.3 (5), 1 (6), 3 (7), 10 (8), 30 (9), and 100 (10) ng/mL.; Figure S5: Calibration curves of S-OFL in S-OFL monoparamentric LFIA with different concentrations of the OFL-protein conjugate immobilized in the TZ: 0.5 (1), 0.25 (2), and 0.125 (3) mg/mL; Figure S6: Calibration curves of S-OFL in S-OFL monoparamentric LFIA with different AS3 dilutions: 1:25(1), 1:50 (2), and 1:100 (3); Figure S7: Selection of TZ arrangement in the double LFIA (at zero point); Figure S8: Selection of AS3 dilution (indicated in the bottom of test strips) for the double LFIA (at zero point); Figure S9. Images of test strips after the double LFIA in an undiluted milk sample. Numbers on the bottom of test strips correspond to S-OFL and R-OFL concentrations of 0 (1), 0.01 (2), 0.03 (3), 0.1 (4), 0.3 (5), 1 (6), 3 (7), 10 (8), 30 (9), and 100 (10) ng/mL; Figure S10: Images of test strips after the LFIA in milk samples. Numbers on the bottom of test strips indicate S-OFL/R-OFL concentrations of 0.56/12.3 ng/mL (1), 1.7/4.1 ng/mL (2), 0.56/0.41 ng/mL (3), and 1.7/12.3 ng/mL; Table S1: Selection of the LFIA conditions; Table S2: Detection results after different sample preparation regimes.

## Abbreviations

The following abbreviations are used in this manuscript:

AuNPs	Gold nanoparticles
AS	Antiserum
BSA	Bovine serum albumin
CR	cross-reactivity
CZ	Control zone
DAGI	Donkey anti-goat immunoglobulins
DCC	*N*,*N*′-Dicyclohexylcarbodiimide
DLS	Dynamic light scattering
DMF	Dimethylformamide
EDC	1-Ethyl-3-(3-dimethylaminopropyl)carbodiimide
ELISA	Enzyme-linked immunosorbent assay
FQ	Fluoroquinolone
gaba	Gamma-aminobutyric acid
GARI	Goat anti-rabbit immunoglobulins
HRP	Horseradish peroxidase
icELISA	Indirect competitive ELISA
LFIA	Lateral flow immunoassay
LOD	Limit of detection
MAb	Monoclonal antibody
NHS	*N*-hydroxysuccinimide
OD	Optical density
OFL	Ofloxacin
OVA	Ovalbumin
PAb	Polyclonal antibody
PBS	50 mM phosphate-buffered saline containing 100 mM NaCl
PBST	PBS containing 0.05% Triton X-100
PBS_TW1_	PBS containing 1% Tween-20
Pdi	Polydispersity index
rac	Racemic mixture of isomers
RT	Room temperature
RU	Relative units
SAL	Salbutamol
STI	Soybean trypsin inhibitor
TMB	3,3′,5,5′-Tetramethylbenzidine
TEM	Transmission electron microscopy
TZ	Test zone
WR	Working range of detectable concentrations
