# Supplementary material for: Double Lateral Flow Test System for Simultaneous Immunodetection of Enantiomeric Forms of Antibiotics: An Ofloxacin Case Study
Source: Biosensors (Basel). 2025 Nov 21;15(12):765. doi: 10.3390/bios15120765 (PMC12730322; doi:10.3390/bios15120765)
Supplement: Supplementary file 1 [file biosensors-15-00765-s001.zip › biosensors-3964039-supplementary.pdf]

biosensors

MDPI

Article

Double Lateral Flow Test System for Simultaneous Immunodetection of Enantiomeric Forms of Antibiotics: An Ofloxacin Case Study

Olga D. Hendrickson, Nadezhda A. Byzova, Anatoly V. Zherdev, and Boris B. Dzantiev*

A.N. Bach Institute of Biochemistry, Research Center of Biotechnology of the Russian Academy of Sciences, Leninsky Prospect 33, 119071 Moscow, Russia; odhendrick@gmail.com (O.D.H.); nbyzova@inbi.ras.ru (N.A.B.); zherdev@inbi.ras.ru (A.V.Z.), dzantiev@inbi.ras.ru (B.B.D.)
* Correspondence: dzantiev@inbi.ras.ru; Tel.: +7-495-954-31-42

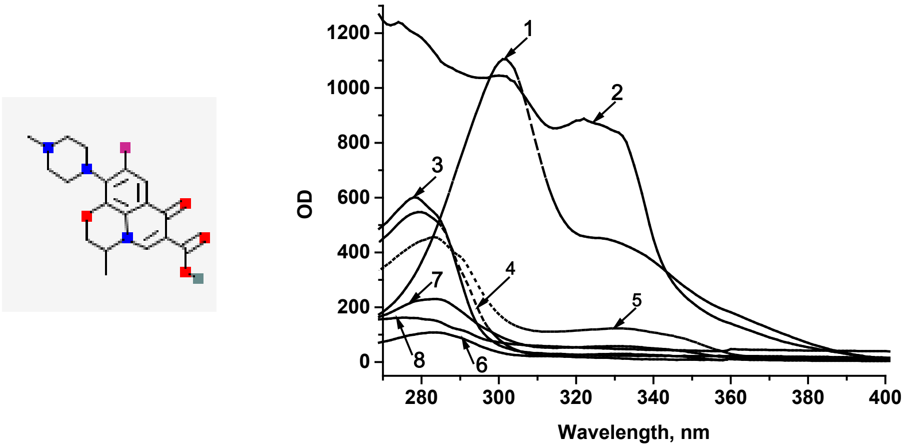

(**a**) (**b**)

Figure S1. Structure of the OFL molecule (from https://pubchem.ncbi.nlm.nih.gov) (**a**) and UV-Vis spectra of S-OFL (1), R-OFL (2), BSA (3), OVA (4), rac-OFL–STI (5), S-OFL-gaba-OVA (**b**), R-OFL-gaba-BSA (7), and STI (8) (b).

Received: 17 October 2025
Revised: 14 November 2025
Accepted: 17 November 2025
Published: 21 November 2025

Citation: Hendrickson, O.D.; Byzova, N.A.; Zherdev, A.V.; Dzantiev, B.B. Double Lateral Flow Test System for Simultaneous Immunodetection of Enantiomeric Forms of Antibiotics: An Ofloxacin Case Study. *Biosensors* **2025**, *15*, 765. https://doi.org/10.3390/bios15120765

Copyright: © 2025 by the authors. Submitted for possible open access publication under the terms and conditions of the Creative Commons Attribution (CC BY) license (https://creativecommons.org/licenses/by/4.0/).

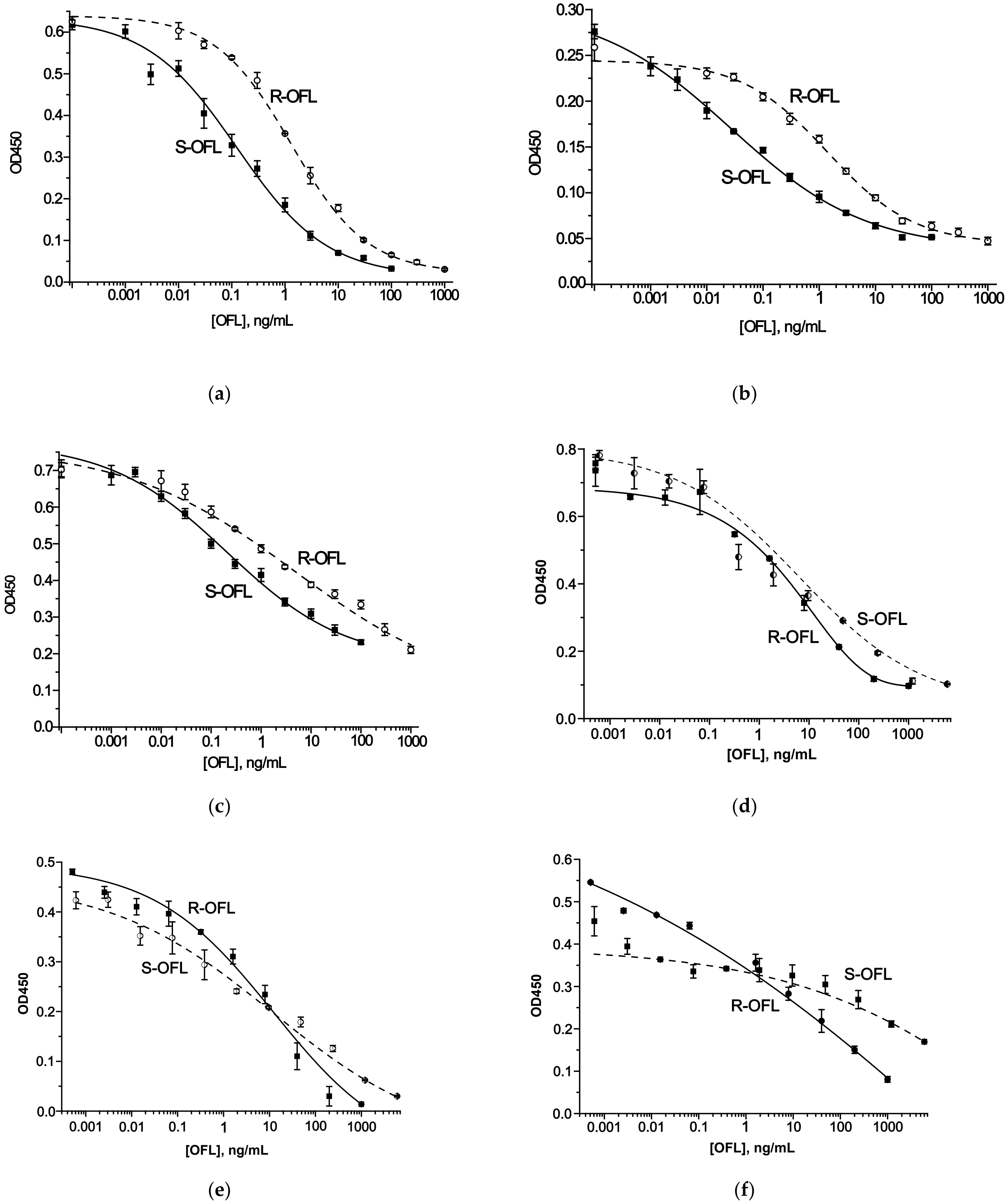

Figure S2. Calibration curves of S-OFL and R-OFL in the icELISA carried out for pre-selection of antiserum for further studies using AS1 (**a**, **d**), AS2 (**b**, **e**), and AS3 (**c**, **f**) and S-OFL-gaba-OVA (**a**-**c**) and R-OFL-gaba-BSA (**d**-**f**) immobilized conjugates.

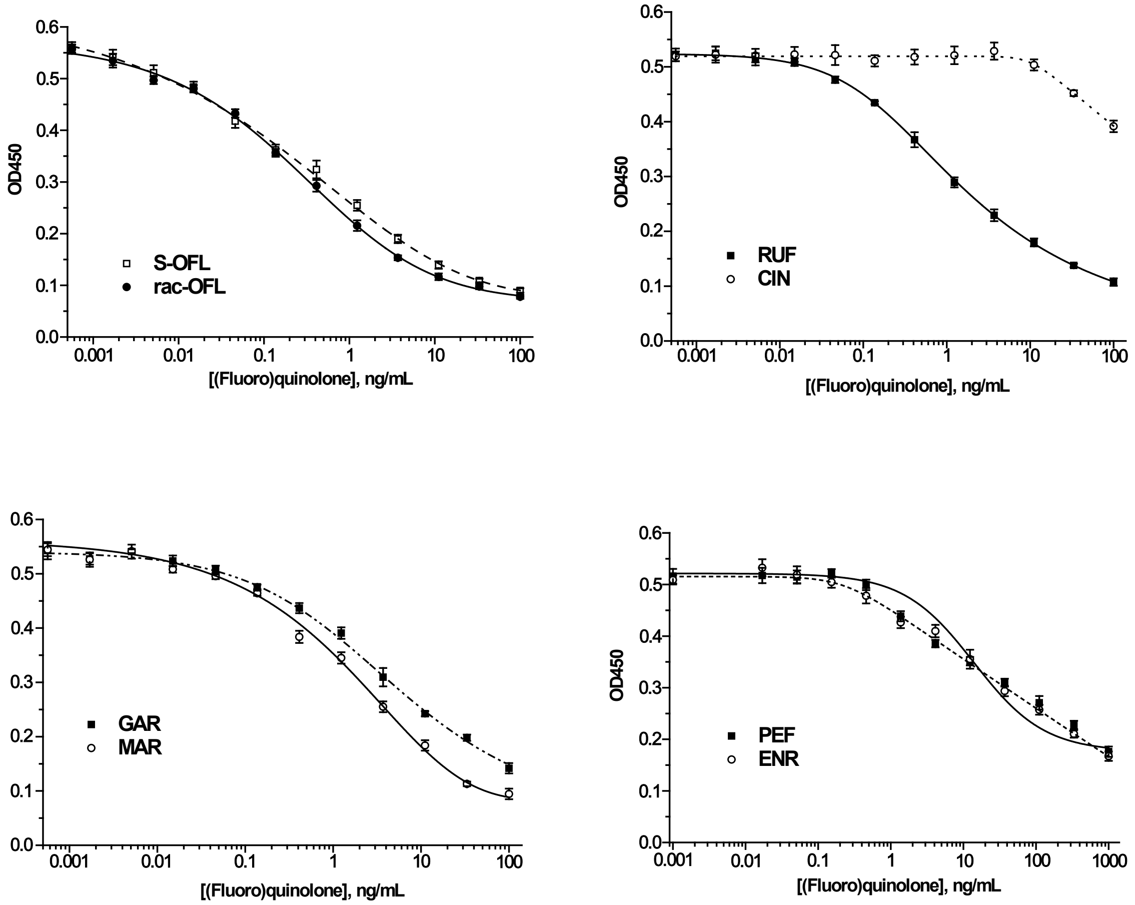

Figure S3. Calibration curves of (fluoro)quinolones cross-reacted with anti-rac-OFL-STI PAb in the ELISA.

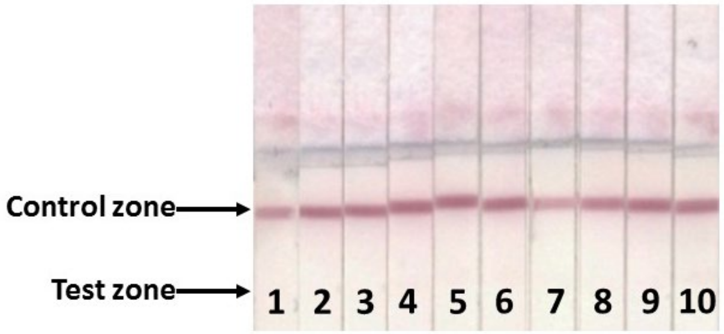

Figure S4. Images of test strips without washing after stage 2 (on the example of monoparametric LFIA of S-OFL). Numbers at the bottom of test strips correspond to S-OFL concentrations of 0 (1), 0.01 (2), 0.03 (3), 0.1 (4), 0.3 (5), 1 (6), 3 (7), 10 (8), 30 (9), and 100 (10) ng/mL.

Table S1. Selection of the LFIA conditions.

Varied parameter	Type/Diapason	Selected parameter
	Monoparametric LFIA	

Concentration of the immobilized rac-OFL-STI conjugate, mg/mL	0.125–0.5	0.5
Concentration of the immobilized DAGI, mg/mL	0.02–0.2	0.05
Dilution of AS3	1:25–1:100	1:25
Volume of the GAMI-AuNPs conjugate, μL	2–10	5
OD_{520} of the GAMI-AuNPs conjugate	2–8	4
Volume of the test sample, μL	30–75	40
Duration of stage 1	1–5	3
Duration of stage 2	3–10	5
Duration of stage 3	1–3	3
Duration of the 1st washing, min	1–5	3
Duration of the 2nd washing, min	1–7	5
Double LFIA		
Concentration of the immobilized S-OFL-gaba-OVA/R-OFL-gaba-BSA conjugates, mg/mL	0.25–1/0.125–0.5	0.75/0.45
Concentration of the immobilized DAGI, mg/mL	0.02–0.2	0.05
Dilution of AS3	1:30–1:1920	1:60
Volume of the GAMI-AuNPs conjugate, μL	2–10	5
OD_{520} of the GAMI-AuNPs conjugate	2–8	4
Arrangement of TZs	TZ1 (R-OFL detection) → TZ2 (S-OFL detection); TZ1 (S-OFL detection) → TZ2 (R-OFL detection)	TZ1 (R-OFL detection) → TZ2 (S-OFL detection)

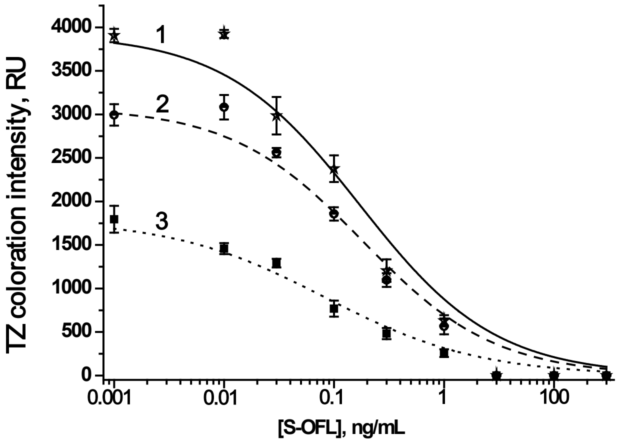

Figure S5. Calibration curves of S-OFL in S-OFL monoparamentric LFIA with different concentrations of the OFL–protein conjugate immobilized in the TZ: 0.5 (1), 0.25 (2), and 0.125 (3) mg/mL.

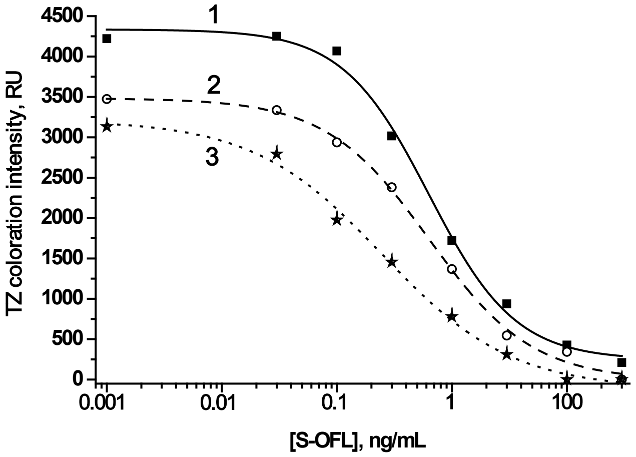

Figure S6. Calibration curves of S-OFL in S-OFL monoparamentric LFIA with different AS3 dilutions: 1:25(1), 1:50 (2), and 1:100 (3).

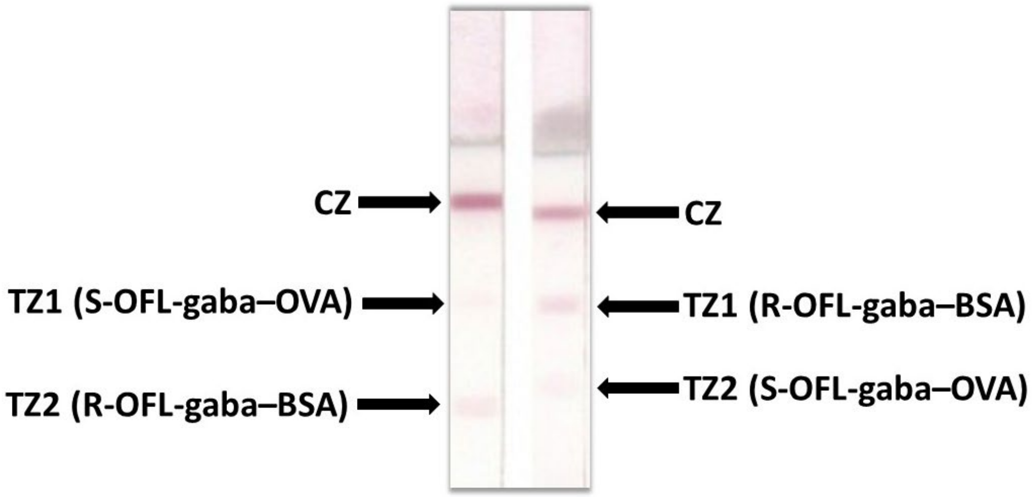

Figure S7. Selection of TZ arrangement in the double LFIA (at zero point).

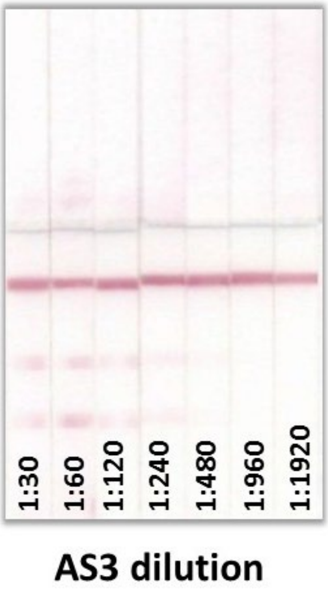

Figure S8. Selection of AS3 dilution (indicated on the bottom of test strips) for the double LFIA (at zero point).

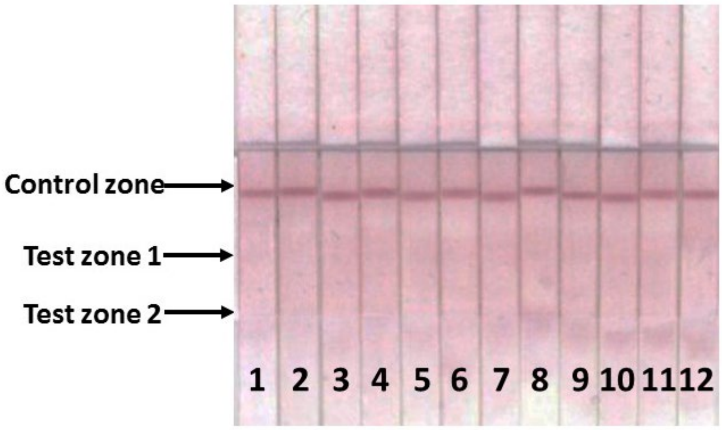

Figure S9. Images of test strips after the double LFIA in an undiluted milk sample. Numbers on the bottom of test strips correspond to S-OFL and R-OFL concentrations of 0 (1), 0.01 (2), 0.03 (3), 0.1 (4), 0.3 (5), 1 (6), 3 (7), 10 (8), 30 (9), and 100 (10) ng/mL.

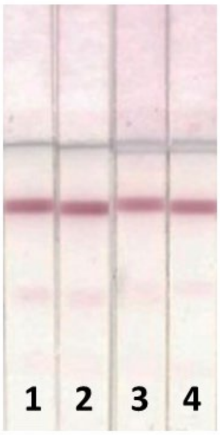

Figure S10. Images of test strips after the LFIA in milk samples. Numbers on the bottom of test strips indicate S-OFL/R-OFL concentrations of 0.56/12.3 ng/mL (1), 1.7/4.1 ng/mL (2), 0.56/0.41 ng/mL (3), and 1.7/12.3 ng/mL.

Table S2. Detection results after different sample preparation regimes.

Milk dilution	Visualization of zones	WR	Sensitivity
Undiluted milk	Impossible	n/p*	n/p*
2-fold diluted milk	Impossible	n/p	n/p
5-fold diluted milk	Excellent	The same as for detection in the buffer	The same as for for detection in buffer
10-fold diluted milk	Excellent	The same as for detection in the buffer	The same as for for detection in the buffer

*not possible to determine
